# Electrochemical properties of roots determine antibiotic adsorption on roots

**DOI:** 10.3389/fpls.2023.930632

**Published:** 2023-03-27

**Authors:** Yuan Liu, Zhen Tao, Hailong Lu, Siyi Li, Chao Hu, Zhongyang Li

**Affiliations:** ^1^ Institute of Farmland Irrigation, Chinese Academy of Agricultural Sciences, Xinxiang, China; ^2^ State Key Laboratory of Soil and Sustainable Agriculture, Institute of Soil Science, Chinese Academy of Sciences, Nanjing, China; ^3^ National Research and Observation Station of Shangqiu Agro-ecology System, Shangqiu, China

**Keywords:** antibiotics, adsorption, legume roots, CEC, zeta potential

## Abstract

The adsorption behaviors and transfer pathways of antibiotics in plant–soil system are greatly influenced by the electrochemical properties of both soil particles and plant roots. However, the effects of roots electrochemical properties on antibiotic adsorption are largely unknown. Here, the fresh soybean, maize, and wheat roots with different electrochemical properties were obtained from hydroponic cultivation, and the adsorption processes and mechanisms of doxycycline, tetracycline, sulfadiazine, and norfloxacin on roots under various environmental conditions were investigated. Results showed that the adsorption amount of antibiotics on roots increased with the initial concentration of antibiotics. The coexisting low–molecular weight organic acids and anions inhibited the antibiotic adsorption on roots. The soybean roots performed strong adsorption ability compared with the maize and wheat roots driven by the variations in root electrochemical properties. This study demonstrates the significance of electrochemical interactions between antibiotics and roots in plant–soil system and can contribute to the more accurate risk assessment and effective pollution control of antibiotics.

## 1 Introduction

Antibiotics, a kind of antimicrobials, have been widely used to treat human and animal diseases and to promote animal growth in animal husbandry and aquaculture. The global antibiotic consumption calculated in defined daily doses was increased by 65% between 2000 (21.1 billion) and 2015 (34.8 billion) and was estimated to increase by 202% in 2030 compared to 2015 if no policy changes (Klein et al., 2018). During these high antibiotic-consuming countries, China ranks first in total consumption (Zhang et al., 2015). The non-metabolized antibiotics by human and animals were excreted out of the organisms *via* urine and feces that could be adopted as low-cost fertilizers for soils (Quaik et al., 2020). Antibiotics can be adsorbed by soil particles, and then, the bioavailable ones can be recruited by the root system of plants (Han et al., 2019) and finally enter into the human body through the food chain, which can lead to an increase in antibiotic-resistant strains, posing threats to human health and ecological environment (Pruden et al., 2006).

Antibiotics, nutrients, and other materials in soil solution enter into the plant *via* the root system. Active functional groups are present in the roots, such as amino groups, galacturonic acid groups, carboxyl groups, and phenolic groups (Meychik and Yermakov, 2001), and their quantities vary from plant to plant. The roots were charged because of the deprotonation or protonation of these groups. In general, leguminous plants are rich in these groups relative to non-leguminous plants. The electrochemical properties of roots affect the physicochemical and biological characteristics of rhizosphere, and all these work together to influence the entry of soil substances into the root system (Jenny and Overstreet, 1939). It has been demonstrated that the electrochemical properties of roots can affect the adsorption and absorption of nutrients and pollutants (Wang et al., 2014; Liu and Xu, 2015; Lu et al., 2018). Antibiotics, a kind of ionic polar organic compounds, generally contain multiple ionic functional groups with different acid dissociation constants (pKa); therefore, the advent of cation, facultative ion, and anion forms are dependent on the medium pH. Logically, we hypothesized that the electrified roots could adsorb antibiotics through electrostatic adsorption and chelation, thus affecting the rhizospheric behavior of antibiotics and the absorption of antibiotics by the roots. In terms of desorption methods, the antibiotics sorbed on the sorbent could be divided into the exchangeable, complex, and residual fraction. CaCl_2_ is usually used to extract the exchangeable fractions (Ye et al., 2016), and dithionite (Na_2_S_2_O_4_, 0.14 mol L^−1^)–sodium citrate (Na_3_C_6_O_7_H_5_, 0.03 mol L^−1^)–sodium bicarbonate (NaHCO_3_, 0.125 mol L^−1^) (DCB) is used for the extraction of the total amount (Yan et al., 2017). However, the studies involved in antibiotic adsorption especially different forms of antibiotics adsorbed on roots under the influence of root electrochemical properties are sporadic.

In general, tetracyclines and quinolones were more easily adsorbed by soil or other medium than sulfonamides due to the discrepancy of functional groups in their molecular structures. Other than pH, ionic strength, and types (cations and inorganic anions) of the coexisting ions played essential roles in antibiotic adsorption or mobility on sand (Li et al., 2019) and also on the particulate organic matter isolated from soils and sediments (Liu et al., 2020). Moreover, the coexisting low–molecular weight organic acids were proved to inhibit the antibiotic adsorption onto biochar (Zhang et al., 2020). However, how these factors impact antibiotic adsorption on roots is little clarified. Many studies have investigated the toxicity of antibiotics to plants affected by the compound types, the compound concentration, the plant species, and the environmental variables (Carvalho et al., 2014; Bartrons and Penuelas, 2017), whereas the mechanisms of toxication associated with adsorption dominated by the root electrochemical characteristics were generally little considered.

The investigation of antibiotic adsorption behaviors on roots is profound for understanding the fate and the environmental risk of antibiotics in farmland soil environment, whereas the relevant research studies are scarce. Hence, wheat, maize, and soybean roots were chosen as the model plant roots, and tetracycline (TC), sulfadiazine (SDZ), doxycycline hydrochloride (DOX), and norfloxacin (NOR) were adopted as the model antibiotics in this study. Our main aims are (1) to expound the influences of environmental variables on the antibiotic adsorption by roots and (2) to demonstrate the essential roles of root electrochemical properties in the adsorption of different forms of antibiotics on the roots.

## 2 Materials and methods

### 2.1 Antibiotics

TC (>98% purity) and SDZ (>98% purity) were purchased from Shanghai Aladdin Biochemical Technology Co., Ltd, and DOX (>98% purity) and NOR (>97% purity) were purchased from Beijing Solarbio Science and Technology Co., Ltd.

### 2.2 Plant materials and cultivation

Fresh roots of soybean [*Glycine max* (L.) Merr., variety Zhonghuang 13 and Xudou 14], maize (*Zea mays* L., variety Jundan 20 and Jinboshi 509), and wheat (*Triticum aestivum* L., variety Bainong 418) were obtained through hydroponic cultivation. Plump seeds were soaked in 10% H_2_O_2_ for 10 min for sterilization followed by the flushing and 4 h of immersing by deionized water. Then, the soybean seeds were cultured at room temperature in sterilized white quartz sand of 0.425 to 0.710 mm with 10% water content and covered by plastic wrap with pre-punctured four small holes to maintain ventilation. The maize and wheat seeds were placed on the gridded baskets in contact with deionized water in the bottom and covered by a lid on the top for germination. On the fourth day, the uniform seedlings were gently pulled out of the sand or the baskets, and the residual endosperm was carefully removed from the maize and wheat seeds using a tweezer. Then, the seedling roots were washed three times with deionized water, transplanted to the 8-L plastic boxes (410 × 240 × 140 mm) filled with nutrient solution, and settled in a growth chamber with temperature of 25°C, day length of 10 h, light intensity of 24,000–28,000 lux, and air moisture of 70%. The ingredients of culture solution were as follows: KNO_3_, 5.0 mmol L^−1^; Ca(NO_3_)_2_·4H_2_O, 5.0 mmol L^−1^; MgSO_4_·7H_2_O, 2.0 mmol L^−1^; KH_2_PO_4_·2H_2_O, 1.0 mmol L^−1^; MnCl_2_·4H_2_O, 10.0 μmol L^−1^; CuSO_4_·5H_2_O, 0.3 μmol L^−1^; ZnSO_4_·7H_2_O, 0.8 μmol L^−1^; Na_2_MoO_4_·2H_2_O, 0.4 μmol L^−1^; H_3_BO_3_, 45.0 μmol L^−1^; and FeSO_4_·7H_2_O-EDTA-2Na, 20.0 μmol L^−^. We renewed the solution every 3 days and conducted the antibiotic adsorption/desorption experiments and the electrochemical properties characterization using the fresh whole roots cut from the seedlings. Here, 20-day-old maize and soybean roots and 40-day-old wheat roots were used to make their root biomass comparable because wheat grows particularly slowly relative to maize and soybean. According to preliminary experiments, roots from these four plants differ highly in cation exchange capacity (CEC) or root surface charges, which could lead to distinct root adsorption of antibiotics (Tai et al., 2018) and thereby the distinct root uptake of antibiotics and the phytotoxicity (Pan and Chu, 2016).

### 2.3 Antibiotic adsorption/desorption experiments

The integrated roots were cut from the base of stems, immediately rinsed by deionized water for 1 min, and then wiped using blotting paper. Afterward, the adsorption experiments begun in 10 min. A nylon bag with 300 mesh was used to hold the fresh roots of approximately 10 g, and then, the bag with the roots was put into a bucket contained with 1 L of antibiotic solution continuously agitated with a magnetic stirrer. In addition, the bag was fixed through a hole in the bucket lid so that the bag was above the magneton (**Figure S1**). The antimicrobial agents NaN_3_ of 0.65 g (to make the final concentration to 0.01 mol L^−1^) were added accompanied with the antibiotics to inhibit the antibiotic degradation by microbes. The TC adsorption was found to reach the equilibrium basically after 0.5 h in the preliminary experiment; hence, 0.5 h was used as the adsorption time for the following adsorption experiments (**Figure S2**). For testing the effects of initial antibiotic concentration, medium pH, coexisting ions, and low–molecular weight organic acids, soybean (Xudou14) roots was adopted as the adsorbent and TC as the test antibiotic. The setup for the adsorption experiments was as follows:

1) Effect of initial antibiotic concentration: 0, 5, and 10 mg L^−1^ were used to ensure the complete dissolution of TC.
2) Effect of medium pH: TC (10 mg L^−1^) with the solution pH values at 4.0, 6.0, 7.36 (CK) (original pH, no adjustment), and 8.0 was used (the solution pH was adjusted three times using NaOH or HCl to get the deprotonation or protonation equilibrium of these groups in TC as much as possible before the adsorption begun).
3) Effect of coexisting cations: TC (10 mg L^−1^) with Ca(II) (CaCl_2_) (1 mmol L^−1^), Mg(II) (MgCl_2_) (1 mmol L^−1^), ${\text{NH}}_{4}^{+}$ (NH_4_Cl) (1 and 2 mmol L^−1^), or Al(III) (AlCl_3_) (0.2 mmol L^−1^) was used. The amount of univalent ion ( ${\text{NH}}_{4}^{+}$ ) (2 mmol L^−1^) was doubled to obtain the equal equivalent with the bivalent ion [Ca(II) and Mg(II)], and the two levels of ${\text{NH}}_{4}^{+}$ ) were used to compare its dose effect on the TC adsorption. The reduced Al(III) concentration of 0.2 mM was selected to reducing the root damage during the contact period due to the high phytotoxicity of Al(III) to the roots (Liu and Xu, 2015) and the remarkable declined root adsorption in this Al(III) concentration in our previous study (Liu et al., 2019).
4) Effects of coexisting anions: TC (10 mg L^−1^) with ${\text{SO}}_{4}^{2-}$ (Na_2_SO_4_), ${\text{SiO}}_{3}^{2-}$ (Na_2_SiO_3_), and ${\text{H}}_{2}{\text{PO}}_{4}^{-}$ (NaH_2_PO_4_) (1 mmol L^−1^) was used.
5) Effects of low–molecular weight organic acids: TC (10 mg L^−1^) with citric acid, malic acid, and oxalic acid (1 mmol L^−1^) was used.
6) Effects of antibiotic types: TCs and DOX, sulfonamides and SDZ, and fluoroquinolones and NOR were used with the initial concentration of 10 mg L^−1^, and maize (Jundan 20 and Jinboshi 509) were used as the model plants.
7) Effects of plant species and variety effects: roots concerning three species and five varieties described above and TC with the initial concentration of 10 mg L^−1^ were used.

The bag, together with the roots, was pulled out of the bucket after 0.5 h of adsorption to finish the adsorption process. Then, the bag containing the roots was immediately immersed in three glass beakers filled with 1 L of deionized water one by one to wash out the residual antibiotics physically adhered on the roots. The blotting paper was applied to remove the attached water on the roots. To desorb the antibiotics on the roots, the bag containing antibiotic-adsorbed roots was laid in 1 L of CaCl_2_ solution (0.01 mol L^−1^) and 1 L of DCB solution (Otte et al., 1991) in sequence for 0.5 h, respectively, in this study to extract the different forms of antibiotics adsorbed on roots. The washing procedure was conducted as mentioned above after each extraction. The concentration of extracted antibiotics in the solution was determined using Liquid Chromatography tandem Mass Spectrometry (LC-MS/MS) (SCIEX Triple Quad 4500, USA)., and the corresponding details were listed in the **Supplementary material**. The desorbed roots were frozen-dried and stored at −80°C until the antibiotic measurement (see details in **Supplementary material**).

### 2.4 Characterization of surface charge of roots

The CEC of roots was determined according to Liu and Xu (2015). For the measurement of zeta potentials, the roots were cut from plants one by one, avoiding being intertwined with each other, carefully washed with deionized water to keep the integrity and alignment of roots, and then air-dried for the measurement of zeta potential. The streaming potential equipment developed by our research team (Li et al., 2015) have successfully measured the zeta potential values of different plant roots (Liu et al., 2016; Liu et al., 2017a; Liu et al., 2017b; Lu et al., 2018; Dong et al., 2020; Lu et al., 2020; Dong et al., 2021; Lu et al., 2021). Details of measurement and data analyses were presented by Lu et al. (2018).

### 2.5 Measurement of root elongation

The root elongation rate (ER) was used as an index of the phytoxicity of antibiotics (Pan and Chu, 2016). The seedlings of 4 days with roots about 3–6 cm were cultured in antibiotic solutions of 10 mg L^−1^ with CaCl_2_ (0.5 mmol L^−1^) for 48 h. The root length at the beginning and the end of the antibiotic exposure was recorded for each seedling. There were 10 repetitions for each treatment. Root elongation per hour was denoted as the root ER.

### 2.6 Statistical analysis

All treatments except the root elongation experiment were performed in triplicate, and the results were reported as means ± standard deviations. One-way analysis of variance (ANOVA) was performed using SPSS 16.0 for Windows (Chicago, USA) for all experiments to compare the significant differences between the treatments. Pearson’s correlation coefficients were determined to examine relationships between the antibiotics adsorbed on roots and root properties. Statistical significance was accepted at *P*< 0.05 in all cases.

## 3 Results

### 3.1 Distribution of antibiotics on roots

#### 3.1.1 Antibiotic concentration effect

**Figure 1:**
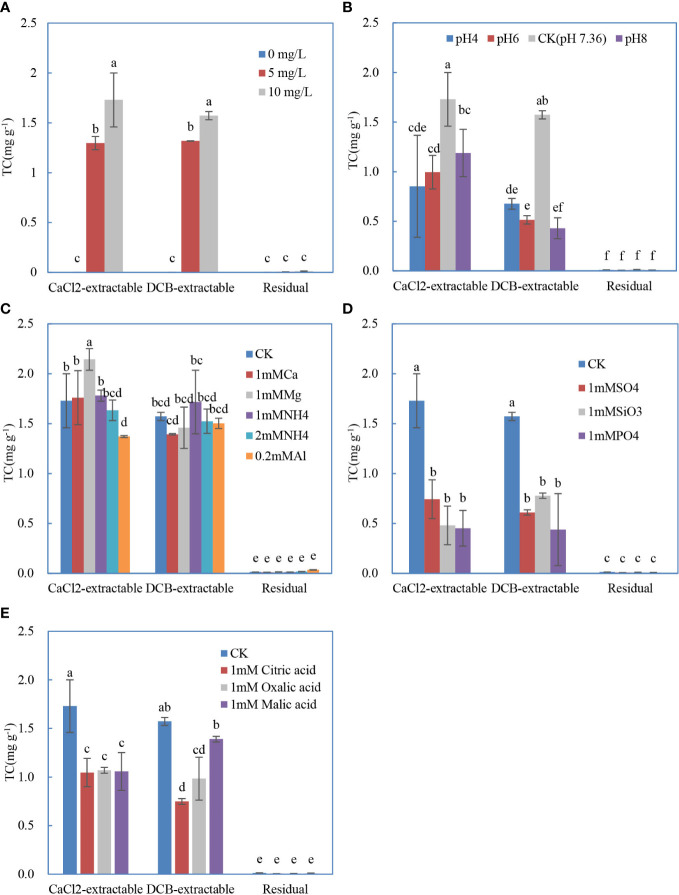
Distribution of the different forms of tetracyline sorbed on the soybean roots influenced by initial concentration **(A)**, initial pH **(B)**, coexisting cations **(C)**, coexisting anions **(D)**, and low–molecular weight acids **(E)**. The data are expressed as the mean ± standard deviation based on three replicates. Lowercase letters above each column denote groups between which significant differences occur at p< 0.05 determined from Duncan’s *post-hoc* pairwise comparisons.

The distribution of CaCl_2_- and DCB-extractable antibiotics on the roots and the residual antibiotics in the roots affected by initial TC concentration is shown in **Figure 1A**. TC was successfully desorbed from roots by CaCl_2_ and DCB, indicating that electrostatic interactions and complexation both drove the TC adsorption on roots, which was consistent with previous studies regarding clay/oxide minerals or other medium (Jia et al., 2008; Wu et al., 2016). The contents of CaCl_2_- and DCB-extractable form were similar and much larger than that of the residual form. With the increase of initial TC concentration, more TC was retained on the roots.

#### 3.1.2 pH effect

As shown in **Figure 1B**, TC solution with the adjusted pH of 8, 6, and 4 prohibited the adsorption of TC significantly compared with the original solution without pH adjustment (pH 7.36). There were no significant differences between the adsorbed TC on roots in solution of pH 8, 6, and 4. The CaCl_2_-extractable form was significantly higher than DCB-extractable form in solution of pH 8 and pH 6. Because the point of zero charges of Xudou 14 root and TC molecular were very close to 4 (Lu et al., 2018) and 6 (Jia et al., 2008), respectively, the CaCl_2_-extractable (electrostatically adsorbed) TC on roots in solutions of pH 4 and pH 6 was rather low. Below pH 7.36, most of TC existed as cationic and zwitterionic species, and its adsorption behavior may resemble that of a cation (Jia et al., 2008). Therefore, the adsorption of TC on the roots increased with the reduction of electrostatic repulsion. At pH > 7.36, the roots carried more negative charges with the increasing pH, and more TC existed as anionic species. Thus, the adsorption of TC on the roots decreased with the increase of electrostatic repulsion. When pH increased from 4 to 6, the positive species TC^+00^ markedly decreased, and, simultaneously, the neutral species TC^+−0^ became the predominant species, the root surfaces bore more negative charges, and the positive surface sites were still far from saturated; thereby, the TC adsorption did not change significantly. As the pH increased from 6.0 to 7.36, TC^+-0^ species declined obviously, whereas the TC^+–^ increased remarkably, and the roots were more negatively charged; therefore, the increase in TC adsorption might be ascribed to the fact that the interaction forces between the negative TC species and the positive sites on roots exceeded the electrostatic repulsion between the negative TC species and roots.

#### 3.1.3 Coexisting cation effects

The cation addition treatments in this study did not significantly change the amounts of the DCB-extractable TC adsorbed on roots compared to that with no addition (**Figure 1C**). For the CaCl_2_-extractable form, only the coexistence of Mg(II) (1 mmol L^−1^) and Al(III) (0.2 mmol L^−1^) significantly altered the TC adsorption on roots, respectively. The presence of Al(III) in the solution suppressed the adsorption of TC probably due to the competition of Al(III) with positive groups of TC for the adsorption sites on root surface, as well as the formation of TC–Al complexes with strong stability. Moreover, Al(OH)_3_ precipitation in the solution could also immobilize part of TC (Chang et al., 2013). On the contrary, Mg(II) amendment enhanced the adsorption of TC, possibly because TC could be adsorbed on the sites where Mg(II) was specifically adsorbed forming TC–Mg(II)–root surface complexes with great stability (MacKay and Canterbury, 2005).

#### 3.1.4 Coexisting anions effects

Unlike cations, all anions significantly declined the CaCl_2_- and DCB-extractable TC on the roots (**Figure 1D**). The adsorbed TC on the roots did not vary with the anion types. Anions (e.g., ${\text{SO}}_{4}^{2-}$ ) were also demonstrated to reduce the TC adsorption on montmorillonite (Wu et al., 2016). The possible reason is that complexes formed between these anions and the positive species of −NH^+^(CH_3_)_2_ in TC(3) (**Figure S3**) were difficult to be adsorbed onto root surfaces. On the other hand, the negative TC^+–^ species were competed by these anions to interact with roots.

#### 3.1.5 Low–molecular weight organic acids effects

In line with the inorganic anions, the addition of low–molecular weight organic acids lowered the adsorption of TC on roots (**Figure 1E**), consistent with the results in the inhibition process of NOR adsorption in variable charge soils (Zhang and Dong, 2008). The lowering effects of these three acids on CaCl_2_-extractable TC were similar, and the impact of malic acid and oxalic acid on DCB-extractable TC was significantly stronger than that of citric acid. Analogously, the dissolved organic matter was also reported to weaken the adsorption of antibiotics onto soils (Zhang et al., 2014). Citric acid, malic acid, and oxalic acid contain polar functional groups, including carboxyl or hydroxyl, which made them to easily interact with the polar fraction of the root surface *via* H bonding and with the hydrophobic fraction by π-π electron donor acceptor interactions (Peiris et al., 2017). The complexation ability order of these three acids is citric acid > oxalic acid > malic acid due to the distinction of molecular structure, which could probably explain the highest reduction in DCB-extractable TC on roots by citric acid. In addition, these organic acids might affect the sorption behavior of antibiotics on roots through competitive sorption or the formation of complexes with antibiotics.

#### 3.1.6 Antibiotic class effects

**Figure 2:**
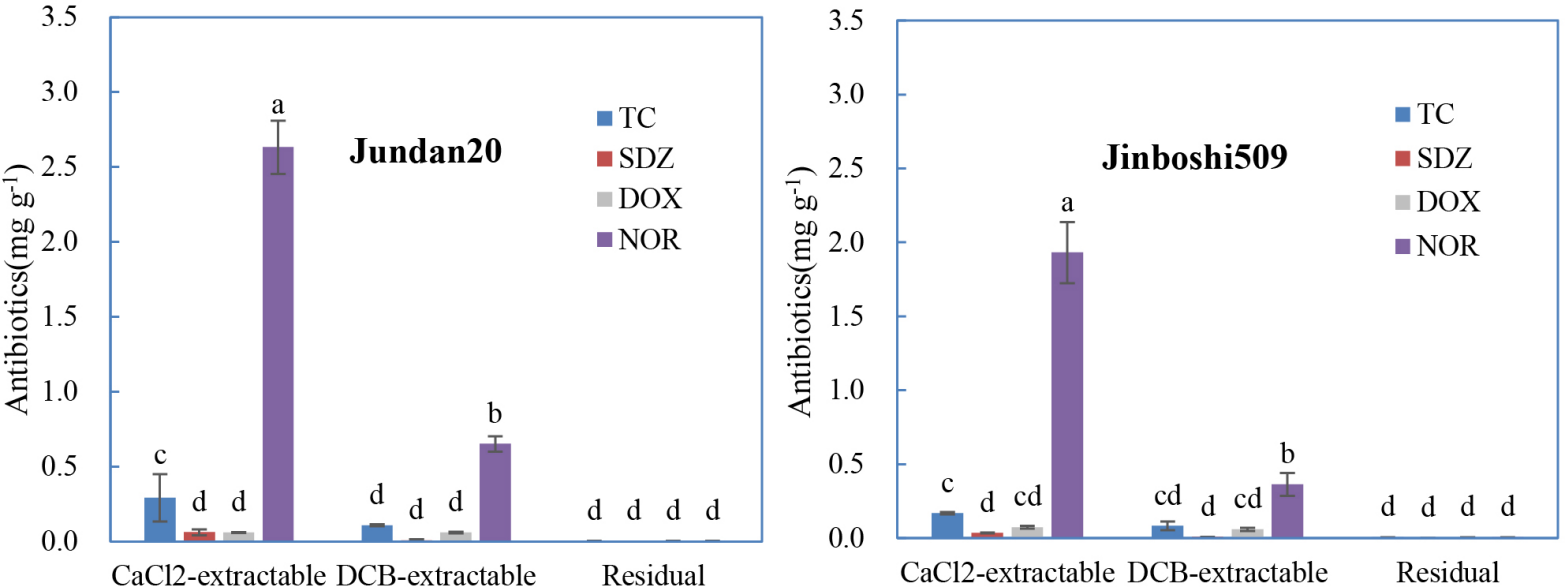
Distribution of the different forms of antibiotics sorbed on the maize roots influenced by antibiotic classes. TC refers to tetracycline, SDZ refers to sulfadiazine, DOX refers to doxycycline, and NOR refers to norfloxacin. The data are expressed as the mean ± standard deviation based on three replicates. Lowercase letters above each column denote groups between which significant differences occur at p< 0.05 determined from Duncan’s *post-hoc* pairwise comparisons.

Four antibiotics belonging to the three classes were used to test the effect of antibiotic classes on antibiotic adsorption on roots. The antibiotics adsorbed on these two varieties of maize roots was similar, although the adsorption was higher on Jundan20 than on Jinboshi509 (**Figure 2**). The CaCl_2_-extractable form of TC was significantly higher than that of DOX for Jundan20 roots. NOR belonging to fluoroquinolones was the most easily adsorbed antibiotics by the maize roots than others, with SDZ belonging to sulfonamides as the least. Taking NOR as an example, the adsorption amount (2.29–3.28 mg g^−1^) on the maize roots in this study was comparable to that (0.297–2.09 mg g^−1^) on the soils (Zhang and Dong, 2008), suggesting that the root adsorption is an indispensable part. In general, the amount of antibiotics adsorbed was impacted by their molecular structures. TC and DOX contain five −OH, two −C=O, one −CONH_2_, and one −N(CH_3_)_2_, and NOR contains one −COOH, one −C=O, and three −N; thus, they can be adsorbed to the adsorbents through electrostatic attraction, cation exchange, cation bridging, complexation, hydrogen bond interaction, among others. Whereas, SDZ containing only one −NH_2_ and one −SO_2_ can be adsorbed mainly by hydrone bridging, cation bridging, hydrogen bonds, and van der Waals forces, which make the distribution coefficients (K_d_) of SDZ much smaller than that of TCs and fluoroquinolones (Thiele-Bruhn, 2003).

#### 3.1.7 Plant species and variety effects

**Figure 3:**
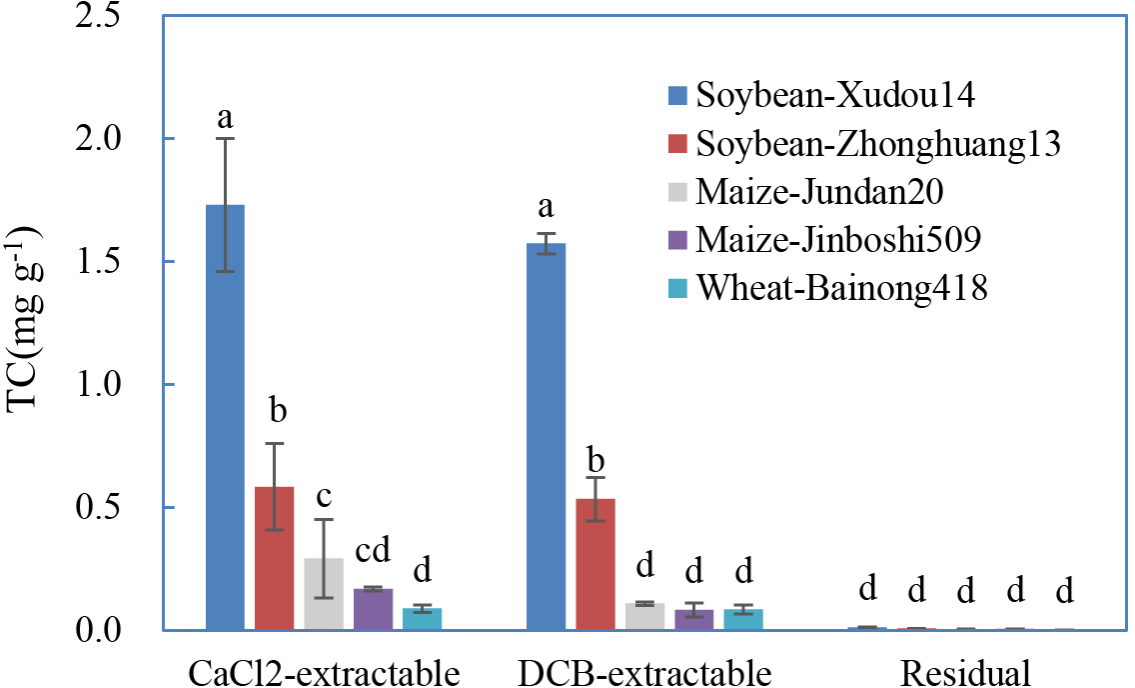
Distribution of the different forms of tetracycline sorbed on the roots influenced by plant species and varieties. The data are expressed as the mean ± standard deviation based on three replicates. Lowercase letters above each column denote groups between which significant differences occur at p< 0.05 determined from Duncan’s *post-hoc* pairwise comparisons.

In general, roots of soybean-Xudou14 and soybean-Zhonghuang13 adsorbed more TC than that of maize-Jundan20, maize-Jinboshi509, and wheat-Bainong418 (**Figure 3**). Except for the interspecific differences, discriminations also existed between plant varieties (Xudou14 *vs*. Zhonghuang13). Roots of legume crops have been deciphered to adsorb more Al(III) (Liu and Xu, 2015) or Mn(II) (Lu et al., 2018) than that of non-legume crops due to the higher negative charge, which is consistent with our results.

### 3.2 Root elongation

**Figure 4:**
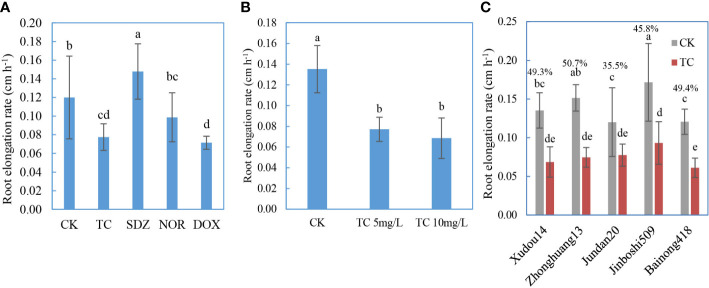
Root elongation rate in antibiotic solution affected by antibiotic classes **(A)**, initial concentration **(B)**, and plant varieties **(C)**. The percentage above the bar **(C)** represents the rate of reduction in root elongation rate affected by antibiotics. TC refers to tetracycline, SDZ refers to sulfadiazine, DOX refers to doxycycline, and NOR refers to norfloxacin. The data are expressed as the mean ± standard deviation based on three replicates. Lowercase letters above each column denote groups between which significant differences occur at p< 0.05 determined from Duncan’s *post-hoc* pairwise comparisons.

The inhibition of root elongation is a valid and sensitive indicator of environmental toxicity (Heberle et al., 2019; Montañés et al., 2020). The effect of antibiotic types on phytotoxicity was presented in **Figure 4A**. After the embrace of Jundan20 roots with these five antibiotics of 10 mg L^−1^, TC, DOX, and NOR suppressed the root elongation significantly, whereas SDZ kind of improved the root elongation. DOX was the most toxic compound although its adsorption by roots was not high (**Figure 2**). The more severe toxicity of TCs than that of fluoroquinolones and sulfonamides to roots was also observed in lettuce, carrot, cucumber, and tomato by a previous study (Pan and Chu, 2016). The root ER was negatively correlated with the CaCl_2_-extractable and DCB-extractable forms of antibiotics adsorbed onto the roots as well as their sum (the total desorption) (**Figure S4**).

The dose response of antibiotics is displayed in **Figure 4B**. TC of 5 mg/L remarkably inhibited the root growth of Xudou14 relative to the control with no TC, whereas the concentration augment from 5 to 10 mg L^−1^ did not cause further retardation of root elongation, although the adsorbed TC increased as shown in **Figure 1A**. Again, the root ER was negatively correlated with the antibiotics adsorbed onto the roots with the R^2^ higher than 0.99 (**Figure S5**).

The phytotoxicity of the same antibiotic compound to different plant species or varieties is exhibited in **Figure 4C**. Compared with the control without TC, TC (10 mg L^−1^) significantly restrained the root growth for all five plant varieties, and the decrease rate of root elongation in TC treatment (relative to the control) was most highest in Zhonghuang13 followed by Bainong418 and Xudou14, and lowest in Jinboshi509 and Jundan20.

### 3.3 Root electrochemical properties

**Figure 5:**
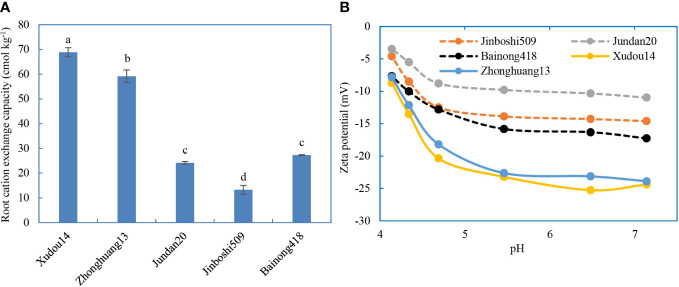
Cation exchange capacity **(A)** and zeta potential **(B)** of roots.

The CEC and zeta potentials (absolute value) of legume roots were significantly higher than those of non-legume roots (**Figure 5**), in accord with the trend of antibiotic adsorption on roots. Significant difference also existed in two soybean varieties. The antibiotics adsorbed on the roots were positively correlated with the CEC of roots (**Figure S6**). Likewise, the inhibition rates of root elongation were weakly positively related with the CEC of roots, suggesting that the roots with higher CEC were more vulnerable to toxicity of antibiotics.

## 4 Discussion

### 4.1 Antibiotic adsorption on roots determines the phytotoxicity of antibiotics

As a whole, the impedance of root growth increased with the increment of antibiotics adsorbed on the roots in our study, and we established the quantitative relationship between the adsorbed antibiotics on roots and root growth rates. The R^2^ values of the linear regression equations based on CaCl_2_-extractable and DCB-extractable forms or the total desorption were very similar, indicating that either form could be used to characterize the hazardous potency of antibiotics to root elongation.

The differential phytotoxicities of TCs to maize and other crops were attributed to their effects on the activities of the crop stress proteins glutathione S-transferases and peroxidases (Farkas et al., 2007). The fluoroquinolones might impair the antioxidative defense system of plants, thereby causing irreversible damage to cellular membranes (Riaz et al., 2017), and inhibit the synthesis of DNA gyrases that catalyze ATP-dependent DNA supercoiling (Liu et al., 2013). Sulfonamides are able to substitute *para*-aminobenzoic acid, which is structurally similar to sulfonamides and involved in a wide variety of metabolic processes of plants including the folate synthesis. Consequently, sulfa-dihydrofolate formed by sulfonamides cannot react with dihydrofolate synthetase, stopping the downstream pathway for folate biosynthesis and resulting in the folate deficiency and poor growth of plants (Prabhu et al., 1997; Cheong et al., 2020). It is known that the effect of SDZ or other antibiotics on plant growth is dose-dependent (hormesis-response). Pan and Chu (2016) confirmed that TC, sulfamethazine, NOR, erythromycin, and chloramphenicol stimulated the root or shoot growth of different vegetables at 0.01 mg L^−1^ and inhibit their growth at higher concentrations. Here, SDZ of 10 mg L^−1^ enhanced the root elongation, indicating that the SDZ level used in this study coincidently enhanced the growth of maize root. Moreover, Michelini et al. (2012) also reported that the root area and fresh mass of maize cultivated in an Orthic Luvisol silt loam spiked with SDZ (10 mg kg^−1^) was significantly increased compared with control despite abnormal root tip geotropism and that the promotion of the accumulation of C and N in stems and leaves by SDZ addition might facilitate the defense of stress. Hormesis refers to a biphasic dose–response to an environmental agent characterized by a low-dose beneficial effect and a high-dose toxic effect. It is a common phenomenon that plants have a hormetic-like response to the antibiotics or phytohormones, which may increase the production of cytoprotective and restorative enzymes including growth factors, thus activating the defense responses of plants (Agathokleous et al., 2018).

### 4.2 The electrochemical properties of roots and antibiotics determine antibiotic adsorption on roots

The former studies have corroborated the significance of electrochemical characteristics of plant roots for the adsorption or uptake of pollutants (Liu and Xu, 2015; Liu et al., 2017b; Lu et al., 2021), nutrients (Meychik et al., 2021), and the corresponding application in evaluating the stress tolerance capability of different plant varieties (Liu et al., 2016; Dong et al., 2021). Here, we confirmed that the root electrochemical characteristics were also essential for the adsorption of antibiotics. Our previous studies showed that the legume and non-legume crops have similar surface functional groups including amide I (antisymmetric stretching vibrations of carboxyl), amide II (N–H bending vibrations), amide III (C–N stretching and N–H bending vibrations), −COOH, C–H, and C–OH (Lu et al., 2018). Meanwhile, the legume roots were always richer in the functional groups than the non-legume crops. The amide, carboxyl, and hydroxyl all have the potential to interact with antibiotics.

Similarly, the properties of antibiotics also affect the electrochemical interaction between antibiotics and roots. Taking TC as an example, it is an amphoteric molecule with multiple ionizable functional groups (**Figure S1**). In aqueous solutions, three different groups of the molecule can undergo protonation-deprotonation reactions depending on the solution pH. The overall charge of TC could be positive (pH< 3.3), neutral (3.3< pH< 7.68), or negative (pH > 7.68) (Jia et al., 2008). At pH 7.36 (the mixture solution of TC of 10 mg L^−1^ and NaN_3_ of 0.01 mol L^−1^) in this study, more negatively charged groups were present on the roots than positively charged groups, and the negatively charged groups were basically comparable relative to the positively charged groups on the TC molecules. As a result, the negatively charged −OH and −CONH functional groups of TC molecules could interact with negatively charged adsorption sites (−COO^−^) on root surface by metal bridging (Chang et al., 2013) (corresponding to DCB-extractable form) and with positively charged amide groups by electrostatic interaction (corresponding to CaCl_2_-extractable form). In this way, it is comprehensible that the coexisting organic and inorganic anions diminished the TC adsorption on roots in our study. The much simpler structure of inorganic anions compared with the organic anions made the less energy-intensive competition with TC, thereby resulting in the more powerful decline of TC adsorption on roots. The log Kow value, the logarithm of octanol/water partition coefficients, is also essential to influence the adsorption ability of antibiotics. The log Kow of these four compounds are as follows: TC (−1.33) (Pan and Chu, 2016), DOX (−0.02) (Kim et al., 2009), NOR (−1.03) (Kim et al., 2009), and SDZ (−0.09) (Carvalho et al., 2014; Ma et al., 2015). The amount of xenobiotic compounds absorbed on roots was positively correlated with the log Kow values of compounds and the total lipid content (the lipid fraction) of the roots (Trapp and Pussemier, 1991). The present study also suggests that log Kow played a role in the phytotoxicity of these antibiotics due to the highest log Kow coupled with the most severe toxicity of DOX. The lipid content distinctions in different plant species might also contribute to the CEC distinctions between plants and, thereby, the antibiotic adsorption differences observed in this study.

Simultaneously, the positively charged −NH(CH_3_)_2_ functional groups of TC could interact with of these negatively charged root adsorption sites through electrostatic interaction or cation exchange (Figueroa et al., 2004). However, the cations except Al(III) did not perform a significant retarding impact on the TC adsorption, possibly because the cation exchange was not the main adsorption mechanism of TC by roots in the scenario of our study. Al(III) ions can powerfully connect with TC and form into potent TC-Al complex, and the complex was difficult to be adsorbed onto roots compared with free TC; consequently, Al(III) ion addition lowered the TC adsorption by roots. In addition, when Mg(II) was added to the solution, it could be adsorbed on roots. The adsorption of TC could take place on the sites where Mg(II) was specifically adsorbed and acted as a bridge between roots and Mg(II), which has been confirmed in other adsorption studies with other organic ligands, such as glyphosate, in the presence of metal ions (Zhou et al., 2004). The differences between Al(III) and Mg(II) were probably caused by the distinctions in the properties of ions themselves and in the properties of their complexes formed with antibiotics, which needs more research in the future. TC contains multiple polar functional groups and could form hydrogen bonding with the polar part of adsorbents (Gu et al., 2007). The combination of these mechanisms enables the adsorption of TC on root surfaces.

### 4.3 Environmental significance

**Figure 6:**
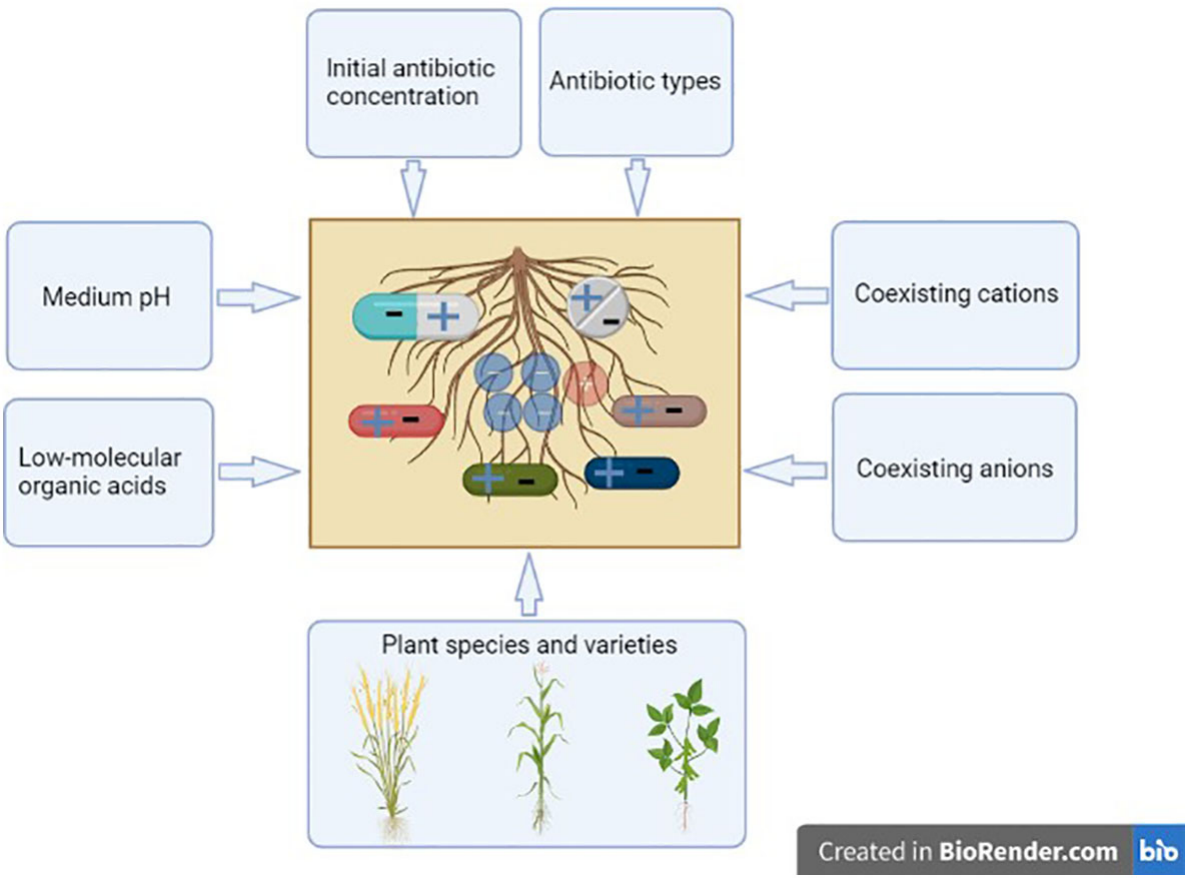
Effects of different factors on the antibiotic adsorption by roots. This figure was created with BioRender.com.

The research findings are of significance to understand the fate and transport of antibiotics in rhizosphere, and the effects of different factors on the antibiotic adsorption on roots are summarized in **Figure 6**. In the antibiotic-polluted soil, plants with roots containing a high CEC could be cultivated for soil remediation. The adsorbed antibiotics on the roots may be desorbed or resorbed by the roots, and, in the soil environment, these processes are also affected by adsorption capacity, temperature, humidity and microbes of soil, light, contact time of antibiotics with roots, root activity, among others. The monitoring of antibiotic distribution in the soil solid phase, soil solution, root surface, root interior, and other plant tissues throughout the growth period merits concern in the future.

## 5 Conclusions

We showed that electrochemical characteristics of roots, which have been unaccounted for in previous studies, can exert a great influence upon the adsorption of antibiotics on roots. Antibiotics could be adsorbed onto plant roots by electrostatic interaction and complexation and other forces and inhibit the root growth. Soybean roots adsorbed more antibiotics than maize and wheat roots due to the higher CEC of soybean roots. The coexisting anions and low–molecular weight organic acids effectively reduced the antibiotic adsorption. Our results implicate that the impacts of root electrochemical characteristics on the behavior of antibiotics especially in rhizosphere warrant more research effort.

## Data availability statement

The original contributions presented in the study are included in the article/**Supplementary material**. Further inquiries can be directed to the corresponding author.

## Author contributions

YL: Conceptualization, Funding acquisition, Supervision, Project administration, Data curation, Visualization, Writing - original draft, review and editing. ZT: Resources, Investigation. HL: Resources, Methodology, Data curation, Formal analysis. SL: Resources, Investigation. CH: Resources, Investigation. ZL: Conceptualization, Supervision, Funding acquisition, Review and Editing. All authors contributed to the article and approved the submitted version.
