# Supplementary material for: Electrochemical properties of roots determine antibiotic adsorption on roots
Source: Front Plant Sci. 2023 Mar 27;14:930632. doi: 10.3389/fpls.2023.930632 (PMC10158730; doi:10.3389/fpls.2023.930632)
Supplement: Supplementary file 1 [file DataSheet_1.docx]

Supplementary material for

**Electrochemical characteristics of roots determine antibiotics adsorption on roots**

Yuan Liu^a^, Zhen Tao^a^, Hailong Lu^b^, Siyi Li^a^, Chao Hu^a^, Zhongyang Li^a,c,*^

^a^ *Institute of Farmland Irrigation, Chinese Academy of Agricultural Sciences, Xinxiang 453002, China*

^b^ *State Key Laboratory of Soil and Sustainable Agriculture, Institute of Soil Science, Chinese Academy of Sciences, Nanjing, 210008 China*

^c^ *National Research and Observation Station of Shangqiu Agro-ecology System, Shangqiu 476000, China*

*Corresponding author. E-mail address: lizhongyang1980@163.com.

Figure captions

Fig. S1 Schematic diagram of adsorption experiment.

Fig. S2 Adsorption kinetics of tetracycline (initial concentration 10 mg L^-1^) by soybean (Xudou14) roots.

Fig. S1 Structure of tetracycline, doxycycline, sulfadiazine and norfloxacin molecule.

Fig. S2 Correlation between root elongation rate and antibiotics absorbed onto maize (Jundan20) roots when comparing the antibiotic class effect.

Fig. S3 Correlation between root elongation rate and the initial tetracycline concentration as well as between root elongation rate and antibiotics absorbed onto soybean (Xudou14) roots when comparing the initial antibiotic concentration effect.

Fig. S4 Correlation between root CEC and the antibiotics absorbed onto roots as well as between root CEC and root elongation rates when comparing the plant variety effect.

**Analysis of antibiotic content in root system**

**Analysis of antibiotic content in solution**


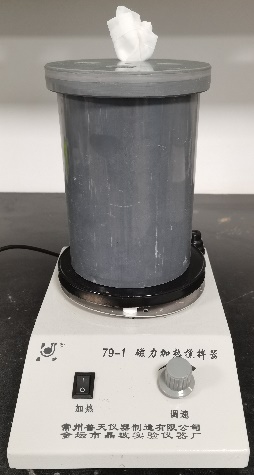


Fig. S1


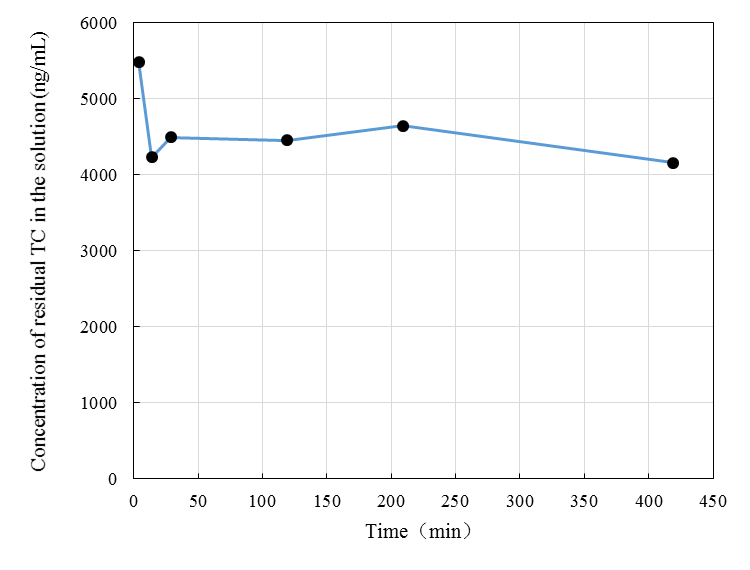


Fig. S2


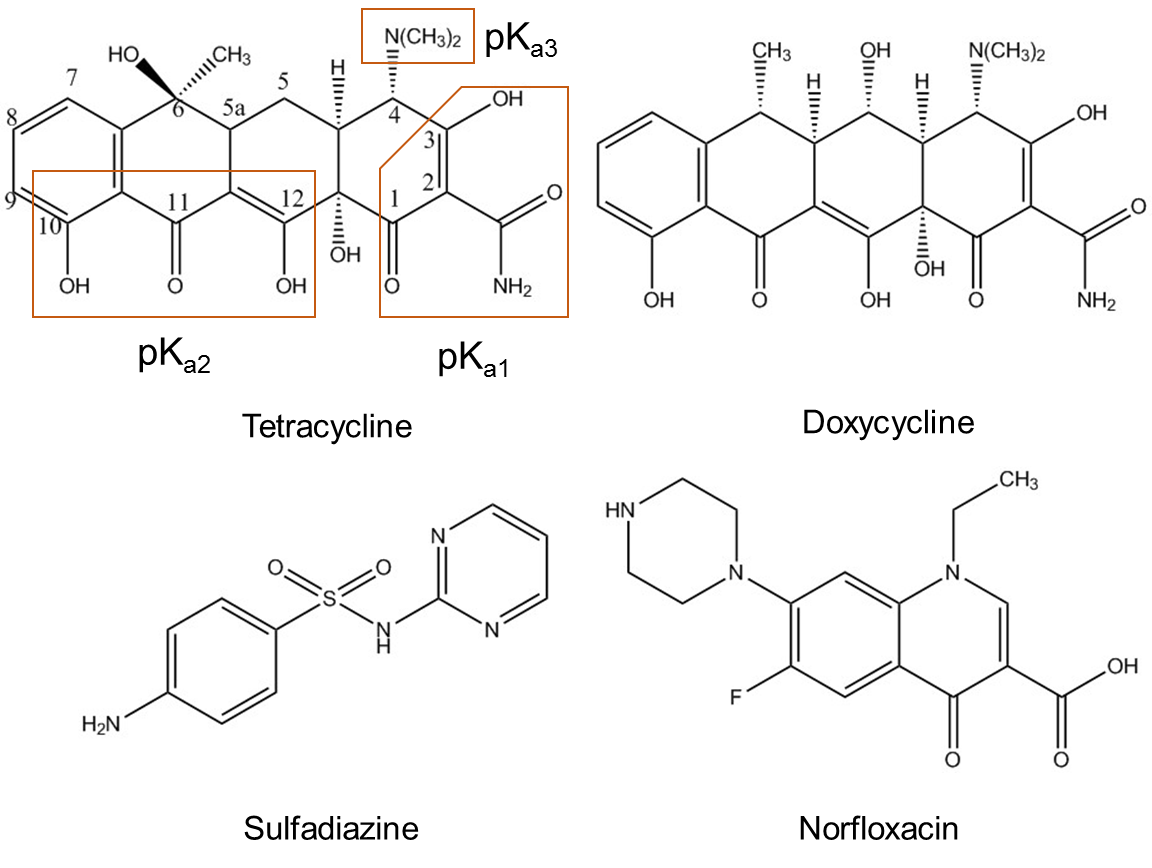


Fig. S1

Fig. S2

Fig. S3

Fig. S4

***Analysis of antibiotic content in root system***

Lyophilized root (<2 g) was mixed with 4 mL Na_2_EDTA-McIIvaine solution (7.1 g sodium hydrogen phosphate, 1.95 g disodium ethylenediaminetetraacetic acid dehydrate and 8.4 g citric acid dissolved in 650 mL deionized water) in a centrifuge tube (50 mL) using a vortex mixer for 1 min, the mixture was blended a second time for 1 min. following addition of 6 mL acetonitrile. Extraction was assisted by ultrasonication for 15 min, followed by centrifugation at 10,000 rpm at 4 ºC for 10 min. All supernatant was transferred to a centrifuge tube (50 mL) containing an extraction salt mixture (4 g anhydrous sodium sulfate, 1 g sodium chloride), the mixture was blended for 1 min, allowed to stand for 10 min until the solution layers were separated, and centrifuged at 10,000 rpm at 4 ºC for 10 min. The acetonitrile layer was taken for analysis. Residual root was extracted using 4 mL methyl alcohol and ultrasonication for 15 min, then centrifuged at 10,000 rpm at 4 ºC for 10 min. The supernatant was added to the previous acetonitrile fraction and 6 mL of the mixture was blended with a purification salt mixture (50 mg *N*-propylethylenediamine solid phase adsorbent, 150 mg octadecyl silicone and 900 mg anhydrous sodium sulfate) for 1 min, centrifuged at 10,000 rpm at 4 ºC for 5 min, dried under gentle nitrogen gas and then diluted to a volume of 1 mL with methanol:water (8:2, vol:vol), blended for 5 min followed by ultrasonication for 5 min, and filtered with 0.22-μm membrane. Finally, the treated samples were analyzed by Liquid Chromatography tandem Mass Spectrometry (LC-MS/MS) (SCIEX Triple Quad 4500, USA).

***Analysis of antibiotic content in solution***

Desorption solution of 2 mL was mixed with 2 mL Na2EDTA-McIIvaine solution in a centrifuge tube (50 mL) using a vortex mixer for 1 min, following addition of 8 mL acetonitrile:methanol (3:1, vol:vol). Extraction was assisted by ultrasonication for 15 min, followed by centrifugation at 10,000 rpm at 4 ºC for 10 min. All supernatant was transferred to a centrifuge tube (50 mL) containing an extraction salt mixture (4 g anhydrous sodium sulfate, 1 g sodium chloride), the mixture was blended for 1 min, allowed to stand for 10 min until the solution layers were separated, and centrifuged at 10,000 rpm at 4 ºC for 10 min. Supernatant of 4 mL were placed to 10 mL centrifuge tube and dried under gentle nitrogen gas and then diluted to a volume of 1 mL with methanol:water (8:2, vol:vol), blended for 5 min followed by ultrasonication for 5 min, and filtered with 0.22-μm membrane. Finally, the treated samples were analyzed by LC-MS/MS.
